# Association between Periodontal Disease and Peptic Ulcers among Japanese Workers: MY Health Up Study

**DOI:** 10.5539/gjhs.v4n2p42

**Published:** 2012-03-01

**Authors:** Chie Kaneto, Satoshi Toyokawa, Kazuo Inoue, Mariko Inoue, Toshihiko Senba, Yasuo Suyama, Yuji Miyoshi, Yasuki Kobayashi

**Affiliations:** Department of Public Health, Graduate School of Medicine, University of Tokyo 7-3-1 Hongo, Bunkyo-ku, Tokyo 113-0033, Japan E-mail: chie-kaneto@umin.ac.jp; Department of Public Health, Graduate School of Medicine, University of Tokyo 7-3-1 Hongo, Bunkyo-ku, Tokyo 113-0033, Japan E-mail: t-satoshi@umin.ac.jp; Department of Community Medicine, Chiba Medical Center, Teikyo University School of Medicine 3426-3 Anesaki, Ichihara-shi, Chiba 299-0111, Japan E-mail: inouek@med.teikyo-u.ac.jp; Department of Hygiene and Public Health, Teikyo University School of Medicine 2-11-1 Kaga, Itabashi-ku, Tokyo 173-8605, Japan E-mail: inoue-ph@med.teikyo-u.ac.jp; Department of Public Health, Graduate School of Medicine, University of Tokyo 7-3-1 Hongo, Bunkyo-ku, Tokyo 113-0033, Japan E-mail: senba-tky@umin.net; Meiji Yasuda Life Foundation of Health and Welfare 1-8-3 Nishi-Shinjuku, Shinjuku-ku, Tokyo 160-0023, Japan E-mail: y_suyama@my-zaidan.or.jp; Division of Health Promotion, Meiji Yasuda Life Insurance Company 2-1-1 Marunouchi, Chiyoda-ku, Tokyo 100-0005, Japan E-mail: yu-miyoshi@meijiyasuda.co.jp; Department of Public Health, Graduate School of Medicine, University of Tokyo 7-3-1 Hongo, Bunkyo-ku, Tokyo 113-0033, Japan Tel: 81-3-5841-3494 E-mail: yasukik@m.u-tokyo.ac.jp

**Keywords:** Periodontal diseases, Periodontitis, Tooth loss, Peptic ulcer, Stomach ulcer, Duodenal ulcer

## Abstract

**Objective:**

This study aimed to investigate the association between periodontal disease and peptic ulcers in a working population.

**Methods:**

Self-administered questionnaires were distributed to all employees of a large insurance company in Japan. The questionnaire asked about their health status and lifestyle habits. Peptic ulcer was defined as either stomach ulcer, duodenal ulcer, or both. For the evaluation of periodontal disease, three indices were used: (a) loss of five or more teeth, (b) having been told of having periodontitis, and (c) periodontal risk score.

**Results:**

Of the eligible 28 765 subjects analyzed, peptic ulcer was present in 397 (1.4%). The results of bivariate analyses showed that a significantly higher proportion of subjects with peptic ulcer reported that they lost five or more teeth (35.3 vs. 17.4%, *p*<0.001) or that they were told they had periodontitis (33.5 vs. 20.7%, *p*<0.001). Moreover, the periodontal risk score was higher for those with peptic ulcer than those without (mean 0.83 vs. 0.59, *p*<0.001). In multivariate logistic regression analyses, statistical associations were found between the presence of peptic ulcer and loss of five or more teeth (odds ratio (OR): 1.41, 95% confidence interval (CI): 1.13–1.76, *p*<0.01), having been told of having periodontitis (OR: 1.28, 95% CI: 1.03–1.59, *p*<0.05), and a 1-point increase in the periodontal risk score (OR: 1.17, 95% CI: 1.04–1.30, *p*<0.01), respectively.

**Conclusion:**

Modest but statistically significant associations were found between the self-reported measures of periodontal disease and peptic ulcers.

## 1. Introduction

Periodontal disease is a group of disorders characterized by infection and inflammation of the tissues surrounding and supporting the teeth (Pihlstrom, Michalowicz, & Johnson, 2005). Gingivitis, the mildest form of periodontal disease, is often caused by inadequate oral hygiene and is readily reversible with good oral care. However, if untreated, gingivitis can progress to more severe periodontitis, which can lead to loosening and eventual loss of teeth (Pihlstrom *et al.*, 2005).

In recent years, the link between periodontal disease and systemic diseases has drawn increasing attention from researchers and clinicians worldwide. Considerable research effort has been focused on the possible role of periodontal disease in the pathogenesis of several systemic diseases, such as cardiovascular disease (Janket, Baird, Chuan, & Jones, 2003; Kuo, Polson, & Kang, 2008; Persson & Persson, 2008), diabetes (Kuo *et al.*, 2008; Taylor & Borgnakke, 2008), adverse pregnancy outcomes (Agueda, Ramón, Manau, Guerrero, & Echeverría, 2008; Wimmer & Pihlstrom, 2008), respiratory diseases (Azarpazhooh & Leake, 2006; Kuo *et al.*, 2008), osteoporosis (Kuo *et al.*, 2008; Wactawski-Wende, 2001), and several kinds of cancers (Abnet *et al.*, 2005; Hujoel, Drangsholt, Spiekerman, & Weiss, 2003; Michaud, Liu, Meyer, Giovannucci, & Joshipura, 2008).

In addition to these diseases, some researchers have suggested that peptic ulcers, including stomach and duodenal ulcers, might also be associated with periodontal disease. A case-control study (Namiot, Namiot, Kemona, & Gołebiewska, 2006) and three cross-sectional studies (including a cross-sectional analysis of baseline data in a cohort study) (Abnet *et al.*, 2005; Khader, Rice, & Lefante, 2003; Molloy, Wolff, Lopez-Guzman, & Hodges, 2004) have examined the relationship between periodontal disease and peptic ulcers; however, no clear conclusions have been reached so far.

In this large-scale cross-sectional study of a working population in Japan, we investigated the association between periodontal disease and peptic ulcers using a self-administered questionnaire.

## 2. Methods

### 2.1 Subjects and the Questionnaire

The subjects of the study were employees of a large insurance company in Japan. The MY Health Up Study was set up in 2004 as part of a health promotion project at the company (Inoue *et al.*, 2007; Inoue *et al.*, 2010; Senba *et al.*, 2008). In October 2004, we distributed self-administered questionnaires to all employees of the company, asking about their health status (physical, mental, and oral) and lifestyle habits. A total of 43 064 questionnaires were distributed, and 34 921 were returned (response rate: 81.1%).

The dependent variable in this study was the self-reported presence/absence of peptic ulcer under treatment. Peptic ulcer was defined as either stomach ulcer, duodenal ulcer, or a combination of both. As for the main independent variable, periodontal disease, we constructed three evaluation indices from the items on the questionnaire: (a) loss of five or more teeth, (b) having been told of having periodontitis, and (c) “periodontal risk score” defined as the number of “yes” answers to the three specific questions about periodontal health; that is, bleeding from the gums, loosening of the teeth, and having been told of having bad breath. Furthermore, we also included in our analysis several other variables possibly related to peptic ulcers or periodontal disease, namely, age, sex, body mass index (BMI), cigarette smoking, alcohol consumption, hypertension under treatment, diabetes under treatment, job stress, and tooth brushing. Among these variables, age, BMI, and the frequency of tooth brushing (per day) were continuous variables; all the others were categorical variables. Smoking status was defined as never-smoker, ex-smoker, and current smoker. Alcohol consumption was dichotomized into “drinking alcohol less than four days a week” vs. “drinking alcohol four days a week or more.” Hypertension and diabetes under treatment were self-reported by the subjects. Job stress was measured in a “job-stress score,” calculated based on the same questions as in the Brief Job Stress Questionnaire (Shimomitsu & Odagiri, 2004). The score ranges from 1 to 5, with a higher score indicating higher job stress.

We excluded from our analysis those who did not answer the questions related to the above variables. In addition, we also excluded those aged 66 and over, because most workers in Japan retire by age 65, as well as those with BMI less than 10 or over 50, because it was likely that some errors have occurred with these subjects.

### 2.2 Statistical Analysis

We first conducted bivariate analyses (Student’s *t*-tests or chi-square tests) to compare the independent variables between subjects with and without peptic ulcers. Then, for each of the three evaluation indices, we calculated odds ratios (ORs) and 95% confidence intervals (CIs) for having peptic ulcers, adjusting for age and sex. We also performed multivariate logistic regression analyses to examine the association between the presence of peptic ulcer and all the independent variables. We used three models; each model included one of the three evaluation indices of periodontal disease (i,e., loss of 5 or more teeth in Model 1, having been told of having periodontitis in Model 2, and periodontal risk score in Model 3) as well as age, sex, BMI, cigarette smoking, alcohol consumption, hypertension under treatment, diabetes under treatment, job stress, and tooth brushing.

In both bivariate and multivariate analyses, a *p*-value of less than 0.05 was considered statistically significant. All statistical analyses were carried out using SPSS for Windows version 11.5J (SPSS Japan Inc., Tokyo, Japan).

### 2.3 Ethics

This study was approved by the Institutional Review Board of the University of Tokyo (No. 1021).

## 3. Results

We analyzed data from 28 765 employees (male 6 688 (23.3%), female 22 077 (76.7%)) in this study. Of the 28 765 subjects, 397 (1.4%) had peptic ulcer (stomach ulcer 175, duodenal ulcer 158, both 64) at the time they answered the questionnaire. Table 1 summarizes the characteristics of the subjects with and without peptic ulcer. The results of bivariate analyses showed that a significantly higher proportion of subjects with peptic ulcer reported that they lost five or more teeth (35.3 vs. 17.4%, *p*<0.001) or that they were told they had periodontitis (33.5 vs. 20.7%, *p*<0.001). Moreover, the periodontal risk score was higher for those with peptic ulcer than those without (mean 0.83 vs. 0.59, *p*<0.001).

**Table 1:** Characteristics of the subjects with and without peptic ulcer

Variable	Peptic ulcer (-) (n=28 368)	Peptic ulcer (+) (n=397)	P-value
Age, mean±SD	43.0±10.5	49.6±8.7	<0.001
Male, n (%)	6 584 (23.2)	104 (26.2)	0.162
Body mass index, mean±SD	22.6±3.5	23.1±3.7	0.006
Smoking status			<0.001
Never-smoker, n (%)	14 865 (52.4)	140 (35.3)	
Ex-smoker, n (%)	3 156 (11.1)	43 (10.8)	
Current smoker, n (%)	10 347 (36.5)	214 (53.9)	
Drinking alcohol 4 days/week or more at present, n (%)	8 391 (29.6)	140 (35.3)	0.014
Hypertension under treatment, n (%)	2 658 (9.4)	70 (17.6)	<0.001
Diabetes under treatment, n (%)	636 (2.2)	30 (7.6)	<0.001
Job-stress score, mean±SD	3.04±0.49	3.16±0.51	<0.001
Frequency of tooth brushing (per day), mean±SD	2.06±0.65	1.96±0.65	0.003
Loss of 5 or more teeth, n (%)	4 931 (17.4)	140 (35.3)	<0.001
Having been told of having periodontitis, n (%)	5 874 (20.7)	133 (33.5)	<0.001
Periodontal risk score, mean±SD	0.59±0.79	0.83±0.90	<0.001

Note: *P*-values shown in the above table are for *t*-tests or chi-square tests. SD; standard deviation

Table 2 shows that, after adjusting for age and sex, the presence of peptic ulcer was significantly associated with loss of five or more teeth (*p*<0.001), having been told of having periodontitis (*p*<0.01), and a 1-point increase in the periodontal risk score (*p*<0.01), respectively.

**Table 2:** Three evaluation indices of periodontal disease and adjusted odds ratios for having peptic ulcer

Variable	Adjusted odds ratio^а^ (95% confidence interval)
Loss of 5 or more teeth (Reference: No)	1.67 (1.34–2.09)‡
Having been told of having periodontitis (Reference: No)	1.45 (1.17–1.79)†
Periodontal risk score (1-point increase)	1.21 (1.08–1.35)†

а Adjusted for age and sex

† p<0.01

‡ p<0.001

Finally, Table 3 displays the results of multivariate logistic regression analyses. Statistical associations were found between the presence of peptic ulcer and loss of five or more teeth (OR: 1.41, 95% CI: 1.13–1.76, *p*<0.01, Hosmer-Lemeshow chi-square test: *p*=0.13), having been told of having periodontitis (OR: 1.28, 95% CI: 1.03–1.59, *p*<0.05, Hosmer-Lemeshow chi-square test: *p*=0.27), and a 1-point increase in the periodontal risk score (OR: 1.17, 95% CI: 1.04–1.30, *p*<0.01, Hosmer-Lemeshow chi-square test: *p*=0.09), respectively. Besides the indices of periodontal disease, older age (*p*<0.001), current cigarette smoking (*p*<0.001), diabetes under treatment (*p*<0.01), and job stress (*p*<0.001) were also significantly associated with the presence of peptic ulcer.

**Table:** Table 3. Multivariate logistic regression models for predicting the presence of peptic ulcer

Variable	Odds ratio (95% confidence interval)		
	Model 1	Model 2	Model 3
Age (1-year increase)	1.07 (1.06–1.08)‡	1.07 (1.06–1.09)‡	1.07 (1.06–1.09)‡
Male (Reference: Female)	1.19 (0.91–1.54)	1.14 (0.89–1.49)	1.13 (0.87–1.48)
Body mass index (1-kg/m^2^ increase)	1.00 (0.97–1.03)	1.00 (0.97–1.03)	1.00 (0.97–1.03)
Smoking status (Reference: Never-smoker)			
Ex-smoker	1.41 (0.98–2.03)	1.41 (0.98–2.03)	1.42 (0.98–2.04)
Current smoker	2.62 (2.07–3.32)‡	2.70 (2.13–3.41)‡	2.75 (2.18–3.47)‡
Drinking alcohol 4 days/week or more at present (Reference: less than 4 days/week)	1.01 (0.81–1.26)	1.01 (0.81–1.26)	1.00 (0.80–1.25)
Hypertension under treatment (Reference: No)	1.27 (0.96–1.67)	1.27 (0.96–1.67)	1.27 (0.96–1.67)
Diabetes under treatment (Reference: No)	1.95 (1.31–2.91)†	1.93 (1.29–2.88)†	1.97 (1.32–2.94)†
Job-stress score (1-point increase)	1.67 (1.36–2.06)‡	1.67 (1.35–2.05)‡	1.64 (1.33–2.03)‡
Frequency of tooth brushing (per day) (1-time increase)	0.99 (0.84–1.16)	0.98 (0.84–1.15)	0.99 (0.84–1.16)
Loss of 5 or more teeth (Reference: No)	1.41 (1.13–1.76)†	–	–
Having been told of having periodontitis (Reference: No)	–	1.28 (1.03–1.59)*	–
Periodontal risk score (1-point increase)	–	–	1.17 (1.04–1.30)†

* p<0.05

† p<0.01

‡ p<0.001

Note: Each model includes one of the three evaluation indices of periodontal disease (i,e., loss of 5 or more teeth in Model 1, having been told of having periodontitis in Model 2, and periodontal risk score in Model 3) as well as age, sex, body mass index, cigarette smoking, alcohol consumption, hypertension under treatment, diabetes under treatment, job stress, and tooth brushing.

## 4. Discussion

In this large-scale cross-sectional study of a working population in Japan, we demonstrated modest but statistically significant associations between the presence of peptic ulcer and three evaluation indices of periodontal disease (i.e., loss of five or more teeth, having been told of having periodontitis, and the periodontal risk score), after adjusting for several factors that could affect the development and prognosis of peptic ulcers.

Of the eligible 28 765 subjects analyzed, peptic ulcer was present in 397 (1.4%). Currently, *Helicobacter pylori (H. pylori)* infection and non-steroidal anti-inflammatory drug (NSAID) use are reported to be the two major risk factors for peptic ulcer. A meta-analysis conducted by Huang, Sridhar, and Hunt (2002) showed that both *H. pylori* infection and NSAID use independently increased the risk of peptic ulcer and ulcer bleeding, and that the ulcer-inducing effects of those risk factors were synergetic. Other factors such as poor socioeconomic status (Rosenstock, Jørgensen, Bonnevie, & Andersen, 2004) and genetic polymorphism (Gillen & McColl, 2005) might also play roles in the development of peptic ulcer.

To our knowledge, only four studies so far have examined the relationship between various measures of periodontal disease and peptic ulcers (including stomach and duodenal ulcers) (Abnet *et al.*, 2005; Khader *et al.*, 2003; Molloy *et al.*, 2004; Namiot *et al.*, 2006). Among them, two groups of researchers have reported a statistically significant association between periodontal disease and peptic ulcer (Abnet *et al.*, 2005; Molloy *et al.*, 2004). Molloy *et al*. (2004) conducted a chart review of 2,006 patients randomly selected from more than 13 000 active patients attending University of Minnesota dental clinics. They showed that, after adjusting for age, sex, and smoking, self-reported stomach ulcer was significantly more prevalent as the number of missing teeth increased. Moreover, subjects with more severe alveolar bone loss were significantly more likely to have stomach ulcer. Abnet *et al*. (2005) also found a significant association between tooth loss and self-reported peptic or duodenal ulcer among 29 124 Finnish male smokers, after adjusting for age. On the contrary, Namiot *et al*. (2006) found no significant association between periodontal disease and peptic ulcer. The results of their case-control study, conducted in Poland, showed that the number of natural teeth and the periodontal index did not differ significantly between 93 peptic ulcer patients and 93 age- and gender-matched dyspeptic controls. In their study, all 186 subjects were infected with *H. pylori*, and only patients with peptic ulcer not related to NSAIDs were included as cases. Another study, conducted by Khader *et al*. (2003), reported mixed results. In their cross-sectional study of 603 patients attending a dental teaching clinic in northern Jordan, self-reported peptic ulcer was significantly associated with increased clinical attachment level, after adjusting for age, plaque index, brushing, smoking, and some other chronic diseases (i.e., diabetes, hypertension, and allergy). However, no significant association was found between peptic ulcer and probing depth, gingival recession, or tooth loss. Compared with the studies mentioned above, our study had a larger or similar sample size and at the same time used multivariate logistic regression models adjusting for more potential confounding factors.

Several mechanisms have been proposed to explain the possible association between periodontal disease and peptic ulcers. Some researchers have pointed out that the oral cavity could serve as a reservoir for *H. pylori*, and as a potential source of infection and re-infection. *H. pylori* have been detected in dental plaque and saliva (Dowsett & Kowolik, 2003), and the success rate of gastric eradication has been reported to be significantly related to the prevalence of the bacteria in the oral cavity (Miyabayashi, Furihata, Shimizu, Ueno, & Akamatsu, 2000). Moreover, a large cross-sectional study conducted in the United States found that periodontal pockets with a depth of 5 mm or more were significantly associated with increased odds of *H. pylori* seropositivity (Dye, Kruszon-Moran, & McQuillan, 2002). The non-significant association between periodontal disease and peptic ulcers in the study by Namiot *et al*. (2006) may thus be explained by the fact that all of their subjects, both cases and controls, were infected with *H. pylori*. On the other hand, it has been reported that polymorphism in certain genes that mediate inflammatory responses might affect the severity of periodontal disease (Cullinan *et al.*, 2001) and also modify the risk for gastroduodenal diseases (Gillen & McColl, 2005). Periodontal disease and peptic ulcer are both inflammatory diseases, and an individual’s poor capacity to handle inflammation might contribute to the progression of both diseases.

In addition to the evaluation indices of periodontal disease, older age, current smoking, diabetes under treatment, and job stress were significantly associated with the presence of peptic ulcer in our study. The associations between these factors and peptic ulcer have also been examined in other studies. Konturek *et al*. (2003) showed that older age and current smoking, as well as *H. pylori* infection and NSAID use, played major roles in the occurrence of peptic ulcer among 5 967 dyspeptic patients in Poland. As for diabetes, a review by Schimke, Chubb, Davis, Phillips, and Davis (2009) indicated that diabetes increases the risk of complications of peptic ulcer, although studies on the association of diabetes and peptic ulcer itself have produced inconsistent results. The role of psychological stress on the development of peptic ulcer also remains controversial. Jones (2006) suggested in his review that psychological factors might be involved in the pathogenesis of peptic ulcer; however, no studies so far proved a causal relationship.

There are several limitations to our study. First, data on *H. pylori* infection and NSAID use, which are major risk factors for peptic ulcer, were not available in this study. We speculate that the significant association between age and peptic ulcer in the present study might reflect the fact that the prevalence of *H. pylori* infection is higher among older generations (Kusters, van Vliet, & Kuipers, 2006). However, it is unlikely that *H. pylori* infection causes periodontal disease. The second limitation is the cross-sectional nature of the study design. Because periodontal disease and peptic ulcer are both considered chronic diseases, the design of this study could not effectively address temporal relationships. A follow-up study of the study population is now being continued. The third limitation is that the information we obtained from the questionnaire was all self-reported. As for the measures of periodontal disease, we used three self-reported evaluation indices (i.e., loss of five or more teeth, having been told of having periodontitis, and the periodontal risk score), instead of clinical dental examinations and periodontal measurements. Studies conducted to validate self-reported measures of periodontal disease so far reported mixed results (Blicher, Joshipura, & Eke, 2005). Medical conditions (i.e., the presence/absence of peptic ulcer, hypertension, and diabetes) were also self-reported; however, a study conducted in Japan showed that self-reported medical histories were generally accurate among the Japanese workplace population (Wada *et al.*, 2009).

In summary, this study demonstrated modest but statistically significant associations between the presence of peptic ulcer and three evaluation indices of periodontal disease, that is, loss of five or more teeth, having been told of having periodontitis, and the periodontal risk score. Longitudinal studies involving clinical dental examination are necessary to further clarify the association.
